# Integrating Loco-Regional Hyperthermia Into the Current Oncology Practice: SWOT and TOWS Analyses

**DOI:** 10.3389/fonc.2020.00819

**Published:** 2020-06-12

**Authors:** Niloy R. Datta, H. Petra Kok, Hans Crezee, Udo S. Gaipl, Stephan Bodis

**Affiliations:** ^1^Centre for Radiation Oncology KSA-KSB, Kantonsspital Aarau, Aarau, Switzerland; ^2^Department of Radiation Oncology, Cancer Center Amsterdam, Amsterdam UMC, University of Amsterdam, Amsterdam, Netherlands; ^3^Department of Radiation Oncology, Universitätsklinikum Erlangen, Friedrich-Alexander-Universität Erlangen-Nürnberg, Erlangen, Germany

**Keywords:** hyperthermia, radiation therapy, chemotherapy, immunotherapy, radiosensitizer, hyperthermia treatment planning, SWOT analysis, clinical trials

## Abstract

Moderate hyperthermia at temperatures between 40 and 44°C is a multifaceted therapeutic modality. It is a potent radiosensitizer, interacts favorably with a host of chemotherapeutic agents, and, in combination with radiotherapy, enforces immunomodulation akin to “*in situ* tumor vaccination.” By sensitizing hypoxic tumor cells and inhibiting repair of radiotherapy-induced DNA damage, the properties of hyperthermia delivered together with photons might provide a tumor-selective therapeutic advantage analogous to high linear energy transfer (LET) neutrons, but with less normal tissue toxicity. Furthermore, the high LET attributes of hyperthermia thermoradiobiologically are likely to enhance low LET protons; thus, proton thermoradiotherapy would mimic ^12^C ion therapy. Hyperthermia with radiotherapy and/or chemotherapy substantially improves therapeutic outcomes without enhancing normal tissue morbidities, yielding level I evidence reported in several randomized clinical trials, systematic reviews, and meta-analyses for various tumor sites. Technological advancements in hyperthermia delivery, advancements in hyperthermia treatment planning, online invasive and non-invasive MR-guided thermometry, and adherence to quality assurance guidelines have ensured safe and effective delivery of hyperthermia to the target region. Novel biological modeling permits integration of hyperthermia and radiotherapy treatment plans. Further, hyperthermia along with immune checkpoint inhibitors and DNA damage repair inhibitors could further augment the therapeutic efficacy resulting in synthetic lethality. Additionally, hyperthermia induced by magnetic nanoparticles coupled to selective payloads, namely, tumor-specific radiotheranostics (for both tumor imaging and radionuclide therapy), chemotherapeutic drugs, immunotherapeutic agents, and gene silencing, could provide a comprehensive tumor-specific theranostic modality akin to “magic (nano)bullets.” To get a realistic overview of the strength (S), weakness (W), opportunities (O), and threats (T) of hyperthermia, a SWOT analysis has been undertaken. Additionally, a TOWS analysis categorizes future strategies to facilitate further integration of hyperthermia with the current treatment modalities. These could gainfully accomplish a safe, versatile, and cost-effective enhancement of the existing therapeutic armamentarium to improve outcomes in clinical oncology.

## Introduction

Moderate hyperthermia (HT), as defined by the Kadota Forum in 2008, is the elevation of the loco-regional tumor temperature between 39 and 45°C, while the recently accepted range is 40–44°C (1–3). HT is perhaps the oldest form of cancer treatment using cauterization by heat as mentioned in the *Edwin Smith Surgical Papyrus*, dated 5000 bc (4). Hippocrates was convinced that tumors incurable by HT were indeed incurable (5). Significant tumor regressions with complete disappearance following high fever secondary to spontaneous Erysipelas infections, or induced through injections of killed cultures of streptococci and *Bacillus prodigiosus* (Coley's toxin), were documented in the mid-nineteenth and early twentieth centuries (6, 7). However, since the advent of penicillin in 1930, reports of tumor regression became infrequent, as high fevers following bacterial infections became rare.

The quest to unravel the biological rationale behind thermal cytotoxicity started in the mid-twentieth century (8–10). Various *in vitro* and *in vivo* studies have documented the thermoradiobiological basis of HT-induced radiosensitization and enhanced tumor cell cytotoxicity (3, 11–13). With various chemotherapeutic agents, HT exhibits synergistic, additive, or independent interactions (14, 15). In addition, local HT enforces immunomodulation akin to “*in situ* tumor vaccination” through upregulation of the release of heat shock proteins (HSPs) that act as danger signals (12, 16–20).

HT with radiotherapy (RT) and/or chemotherapy (CT) in various tumor sites have been investigated in various clinical trials (21–83). Systematic reviews (12, 84–87) and meta-analyses have reported positive outcome with HT (88–95). An overall complete response (CR) of 54.9% with HTRT vs. 39.8% with RT alone (risk difference = 0.15, 95% CI: 0.11–0.20, *p* < 0.001) was reported from 38 clinical trials of HTRT vs. RT alone in 3,478 patients with various tumor sites (RT, *n* = 1,717; HTRT, *n* = 1,761) (12). Significant developments in hardware and software, treatment planning, and online thermometry have enabled safe and effective delivery of HT (2, 87, 96–105). Moreover, with the increasing understanding of the pathways of molecular interaction of HT with DNA damage repair and also its role in immunomodulation, integrating HT with RT and CT, DNA damage repair inhibitors and/or immune checkpoint inhibitors (ICIs) as may be indicated in a clinical situation could provide a novel approach in contemporary oncology practice (19, 20, 106–111).

In a bid to translate the therapeutic advantages of HT and optimally integrate HT into the oncological therapeutic armamentarium, a SWOT analysis was performed. This allowed a realistic evaluation of the current status of HT in terms of its strength (S), weakness (W), opportunities (O), and threats (T). With the use of the key findings of SWOT, a TOWS analysis (acronym similar to SWOT) was carried out to examine the strengths and opportunities of HT that could be used to address its present weakness and threats and thereby identify future strategies that could facilitate successful integration of HT with other treatment modalities. The review will be restricted to the loco-regional application of moderate HT as a thermal sensitizer adjuvant to RT and/or CT in solid tumors. Thus, hyperthermic chemoperfusion and thermoablative techniques are beyond the scope of this review.

## Hyperthermia: Its Interaction With Other Therapeutic Modalities

Loco-regional HT in the range of 39 and 45°C exerts pleotropic effects in both normal tissue and tumors. Various preclinical *in vitro* and *in vivo* studies have reported on the direct and indirect effects of HT and may be summarized as follows:

- Heat over a temperature range of 41.5–46.5°C kills cells in a predictable, exponential manner; and the rate of killing increases with temperature and is attributed to the denaturation of the various structural proteins of the its organelles and deactivations of the enzymes (112).
- Hyperthermia results in inhibitions of the DNA repair enzymes involved with sublethal and potentially lethal damages, thereby potentiating the effects of RT and CT leading to mitotic catastrophe, induction of senescence, apoptosis, and necrosis (10, 106, 113–115).
- Low pH exerts a profound cytotoxic effect and enhances thermal sensitivity, especially at 41–43°C (116, 117). This would have direct implications in hypoxic regions of tumor, which are usually radioresistant owing to low pH secondary to elevated glycolysis and lactic acid production (118).
- Cells in the “S phase,” which are radioresistant and heat sensitive (10, 119). These effects of HT could act in a complementary manner to RT and/or CT to improve the therapeutic outcomes of a combined intervention with HT.

Thus, with HT, the changes are a result of temperature-dependent effects on loco-regional vascularity, interactions at the molecular levels especially DNA repair, induction of HSPs, modification of the tumor cell phenotype, and direct thermal cytotoxicity (15, 120). Gradual rise of temperature to 39°C and beyond augments loco-regional vascular flow, in both normal and tumor tissues. It has been reported that the alterations in the perfusion following heating are much greater in the normal tissues than in tumors. Following HT, the blood flow could increase by at least a factor of 15 compared with only twice in tumors (121). The extent and the dynamics of alterations in the tumor blood have been found to depend on heating up rate, heating time, homogeneity of achieved intratumoral temperature, tumor type, and site. Longer exposure to temperatures higher than 42.5°C could result in impairment of blood flow due to damage of the fragile and chaotic neoangiogenic tumor microcirculation. This could result in even complete shutdown of tumor blood flow (121–123). The direct thermal toxicity on the tumor increases rapidly beyond a threshold temperature of 42.5–43°C for 60 min, a risk depicted typically using Arrhenius plots (124, 125).

Most preclinical HT research used and uses *in vitro* conditions, where cells cultured are uniformly exposed to a well-controlled predefined temperature, but the situation during clinical HT is generally different. Although every effort is made to reach temperatures of 41–43°C in patients during HT treatment, the temperatures attained usually vary between 39 and 44°C. This depends on the tumor depth, vascularity, and perfusion; specifications of the HT equipment, applied power, and amplitude steering abilities; adjacent normal structures with variable dielectric properties (muscle, bone, air, etc.); loco-regional blood flow; and individual patient tolerance. Thus, achieving a uniform tumor temperature of 41–43°C as in *in vitro* conditions may not be always feasible in clinical situations.

One should therefore be prepared to accept a compulsive heterogeneity in temperature distribution within the tumor and set realistic therapeutic goals (126). Consequently, the dominant temperature-dependent HT effects at molecular and cellular levels would reflect this inevitable thermal diversity within the heated tumor volume. From the immunological point of view, heterogeneous temperatures should be even beneficial, because temperatures of 39°C and higher foster immune cell infiltration and temperatures above 41°C lead to immunogenic cancer cell death. These complementary effects activate the immune system at multiple levels as described later (111).

Translating *in vitro* conditions of a predefined fixed temperature and time may not be always feasible in clinics, nor may this be obligatory. Clinical HT has to adapt to individual patient's tolerance and acceptance. Thus, direct “bench-to-bedside” approaches needs realistic amendment in clinics to deliver temperatures that are achievable and tolerable by patients over the range of 39–45°C. This calls for setting clear goals of HT in clinical application (3, 13, 126). Thus, interaction of HT along with RT, CT, and its immunomodulatory effects is expected to evolve along the temperature range of moderate HT. It would be inappropriate to separate these interactions in water-tight compartments; instead, the effect HT should be considered as a cumulative effect on blood flow changes and oxygenation, DNA repair inhibition, thermal sensitization, and direct thermal cytotoxicity over the temperature range of 39–45°C (120). As these interactions hold key to the thermal potentiation of these therapeutic modalities, a brief summary of the various relevant interactions between HT and these modalities, namely, RT, CT, and immunomodulation, is given below before presenting the SWOT and TOWS analyses.

## Hyperthermia: A Potent Radiosensitizer

Radiation therapy is one of the mainstays of treatment of cancer either alone or in combination with other therapeutic modalities. It is estimated that 45–55% of newly diagnosed cancer patients need RT and an additional 10% patients would need reirradiation in their lifetime (127, 128). Forty percent of the cancer cures are attributed to RT (129). The primary aim of a RT treatment is to deliver a tumoricidal dose to tumors by inducing DNA damage while minimizing the dose to the normal tissues. Thus, a number of advancements in RT treatment delivery, planning, execution, and monitoring have made modern RT both safe and efficacious (130). However, some inherent limitation of RT still remains. These are primarily as follows: (a) intrinsic DNA repair could prevent conversion of sublethal and potentially lethal damages to lethal damages, thereby reducing tumor cell kill; (b) presence of hypoxic tumor cells confers radioresistance to tumor cells; (c) “S” phase tumor cells are intrinsically radioresistant; and (d) escalation of RT dose to enhance tumor control could result in a higher risk of acute and late normal tissue morbidity. Thus, of the various different attempts that have been made to improve the therapeutic outcomes with RT, the search for appropriate radiation sensitizers and radiation modifiers constitutes one of the actively investigated areas (131, 132).

**Figure 1:**
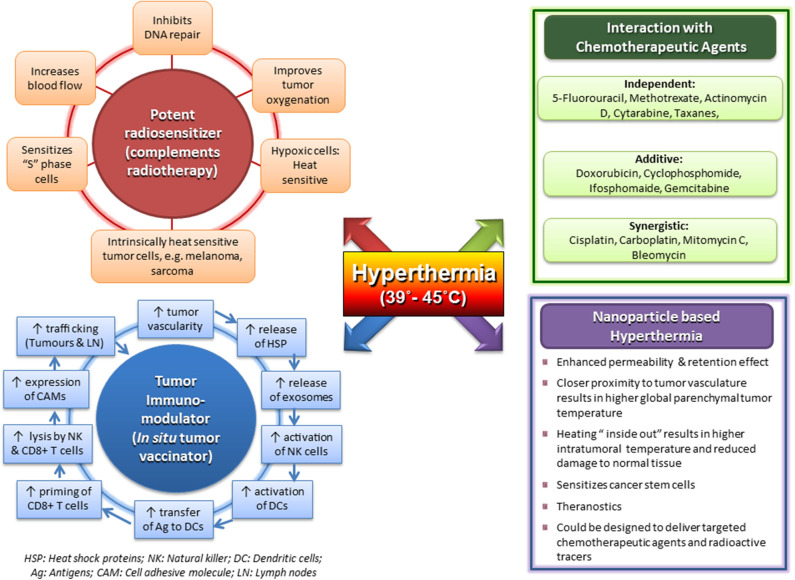
Multifaceted action of clinical hyperthermia at 39–45°C with its effects as a potent radiosensitizer; independent, additive, and synergistic action with chemotherapeutic agents; a tumor immunomodulator with potential as an *in situ* tumor vaccinator; and its prospects using magnetic nanoparticles with payloads. Reproduced with permission from Datta et al. (12).

Moderate HT is one of the most potent known radiosensitizers through a combination of induced thermophysiological changes in the tumor matrix, along with sustainable interactions at the cellular and molecular levels (3, 9, 12, 13, 86, 106–108, 133–145) (Figure 1). The effects of interaction of HT with RT are summarized as follows:

- *Hyperthermic radiosensitization*: Moderate HT radiosensitizes in principle both normal and tumor tissues. The thermal enhancement ratio (TER) quantifying the radiosensitization depends on the tissue type, temperature, heating time, and time interval between heat and irradiation (13). The higher the temperature and the longer the heating period, the greater the enhancement (142, 146). When HT and RT are delivered simultaneously, the TER is the highest with a proportional linear relation with heat dose and may even reach 5-fold enhancement, but it also attains similar values for both tumor and normal tissues (84, 142, 147). Thus, simultaneous HTRT requires RT to precisely conform the tumor target to achieve tumor selectiveness (13). As the time interval between HT and RT increases, irrespective of their sequence, TER drops to plateau at around 2 in tumor tissue, with greater reduction to a TER of around 1 in normal tissues. Consequently, clinically, HT and RT are normally given sequentially, and a short time interval between HT and RT is considered optimal, as it yields both effective and tumor-selective radiosensitization. Tumor selectiveness is further enhanced because temperatures are generally lower in the normal tissue margin around the tumor, yielding lower TER (102).
  Radioresistant late “S” phase cells are heat sensitive. HT also temporarily inhibits the repair of RT-induced sublethal and potentially lethal DNA damage in irradiated cells at temperatures >41°C (10, 106, 114). Clinically, exploiting this mechanism requires both high tumor temperatures and short time intervals between RT and HT before RT-induced DNA damage gets repaired (148). HT inhibits the homologous recombination (HR) repair of DNA double-strand breaks (DSBs) by inducing degradation of BRCA2, a key protein for HR repair, with a clear dose–effect relationship (109, 149). Classical non-homologous end-joining (c-NHEJ) and alternative non-homologous end-joining (alt-NHEJ), the other major DSB repair pathway (118), are also reported to be partly affected as important repair proteins display decreased levels and activity after HT, including Ku, DNA-PK, KU70, KU80, and Ligase IV (106). HT was also demonstrated to be an effective radiosensitizer in BRCA2-deficient tumor cells without active HR, indicating HT-induced blocking of other DNA repair pathways (108). The resulting DNA repair inhibition significantly reduces the tumor α/β values widely used in the linear-quadratic (LQ) model to optimize RT fractionation schedules (114). Thus, hypofractionation strategies, as currently favored in a number of clinical situations (including use of particle therapy), would benefit strongly from adding HT to RT (150).
  Physiological vasodilation following HT also contributes to radiosensitization by improving tumor perfusion and oxygenation, thereby rendering treatment-resistant hypoxic cells radiosensitive. This effect requires relatively low, easily attainable tumor temperatures of around 39°C (122, 126, 151). Clinical data show that HT leads in part of the tumors to reperfusion and reoxygenation (152, 153), and this reoxygenation appears to be associated with better clinical outcome (154, 155). Raising tumor temperatures to 44°C and higher can result in vascular shutdown, reduced tumor oxygenation, and poor clinical outcome as opposed to regions with a milder temperature elevation (156). The enhanced oxygen levels appear to last up to 1–2 days after HT treatment (152, 154). The occurrence of tumor reperfusion depends among others on the ability of the tumor vasculature to dilate in response to elevated temperatures, which is tumor specific (123). These have implications in tumor reoxygenation when HT is used with RT and also with CT for drug delivery within tumors.
- *Hyperthermic cytotoxicity*: Heat kills cells by various mechanisms including necrosis and apoptosis (157–159). In tumors, known to harbor hypoxic cells, HT exerts cytotoxicity to these cells lodged in adverse microenvironment and low pH (13). Thus, HT-induced cytotoxicity is primarily directed toward the chronically hypoxic cell fraction embedded in the acidic milieu (160). This effect is temperature dependent but independent of the time interval between HT and RT (13, 146). HT-induced cytotoxicity would thus help to reduce the nidus of these radioresistant cells and improve clinical outcome.
- *Thermotolerance*: Thermotolerance is induced during an HT session, resulting in a transitory resistance to subsequent HT sessions, thereby influencing the thermal sensitivity of tissues to subsequent HT (84, 124). The phenomenon is attributed to HSPs, which are upregulated following heat stress (125). These are ubiquitous proteins acting as “chaperones” and are involved both in thermal tolerance when being inside the cell and also for immunomodulation when being released. Thermotolerance decays subsequently with temperature-dependent kinetics and is dependent on the initial thermal damage and the intervening time elapsed between the subsequent HT sessions. The higher the initial tumor temperature, the longer the thermotolerance persists (125, 147, 161). Clinically, HT is delivered once or twice a week to avoid ineffective HT sessions due to thermotolerance. In view of the complex interplay of the thermoradiobiological interactions, a pragmatic approach in clinical situation is desirable.

Taking into consideration all the above factors, it might be imperative to define an optimum thermal dose that could be prescribed for thermoradiotherapy (HTRT) treatments. In RT, a specific dose prescription with dose per fraction and overall treatment time to various tumor volumes are mandatory and are judiciously implemented during RT treatment delivery. In case of CT, drug prescriptions are usually based on body weights or surface areas. The amounts of drug finally delivered to the tumor site are unknown, but this has not been a deterrent to routine CT prescriptions. It is well-understood that precise tumor drug dose quantifications are not practically feasible and drug dose modifications are usually based on the toxicity profiles of individual patients. In case of HT, Sapareto and Dewey (162) had proposed a thermal isoeffective dose to convert one-time temperature combination to a standard level, by typically converting the time-temperature data to an equivalent number of minutes at 43°C, the cumulative number of equivalent minutes at 43°C (CEM43°C). Later, this was further modified to CEM43°C T90 to indicate the cumulative number of equivalent minutes at 43°C exceeded by 90% of the temperature points within the tumor (124). This parameter has been shown to directly correlate with the treatment outcome (22, 163). However, consensus was lacking, and a wide range of various temperature and thermal dose parameters have been reported in various clinical trials; readers interested in these issues are referred to the excellent overview by van Rhoon (120).

The recent guideline of the European Society for Hyperthermic Oncology (ESHO) for application of superficial HT has therefore recommended that temperatures achieved in 10, 50, and 90% of measured temperature points should be reported as T10, T50, T90, and the thermal dose as CEM43°C T10, CEM43°C T50, and CEM43°C T90, respectively (2). In addition, the guideline stated to aim for a T90 exceeding 40°C and T50 more than 41°C. The wider use of this recommended thermal dose reporting would ensure quality assurance in HT treatment delivery and assessment. Further, use of uniform reporting standards could provide insights into the thermal dose–response relationship, permitting intercomparison between various studies and also optimizing HT treatment prescriptions.

## Hyperthermia: A Chemosensitizer

Cytotoxicity of a host of CT agents is modified by HT (14, 113, 164, 165). *In vivo* and *in vitro* studies have demonstrated that a number of CT agents exhibit thermal enhancement at 40.5–43°C. Urano et al. (166) have reported that the cell survival curves becomes steeper, indicating thermal sensitization for 1,3-bis(2-chloroethyl)-1-nitrosourea (BCNU), *cis*-diamminechloroplatinum[II] (CDDP), and bleomycin (BLM) as temperatures rise from 37 to 41.5°C, resulting in significant reduction in D_0_. This was not significant for 5-fluorouracil (5-FU) and mitomycin C (MMC), whereas for adriamycin (ADR), this was evident only from 41 to 43°C. The changes in the slopes of D_0_ indicate that the activation energy of these agents increases significantly at higher temperature and thus relates to the subsequent thermal enhancement of drug cytotoxicity (166).

Physiologically heat-induced vasodilation at temperatures of 39–43°C allows enhanced drug delivery to the tumor by improving the blood flow and increasing the vascular permeability. The consequent thermal sensitization of CT has been quantified in terms of a TER and has been estimated to vary from 1.0 to 3.6 (166). Accordingly, the interaction of HT and CT could be broadly classified as independent action (5-FU, methotrexate, actinomycin D, cytarabine, and taxanes), additive (doxorubicin, cyclophosphamide, ifosfamide, and gemcitabine), or synergistic (cisplatin, carboplatin, oxaliplatin, MMC, and BLM) (Figure 1) (14). Although most of the drugs show a linear relation of thermal sensitivity with temperature in the range of 37–40°C, doxorubicin and BLM are reported to have a threshold temperature of around 42.5°C (15). Alkylating agents cisplatin and oxaliplatin show rapidly increasing TER values for temperatures exceeding 41–42°C (167), probably reflecting synergistic action with heat-induced blocking of DNA damage repair, similar to the synergy with RT. Trabectedin induces DNA DSBs and also displays a clear coaction when combined with HT (164, 168). Most of the CT agents are most effective when delivered just before or during HT. However, for gemcitabine, *in vitro* studies in pancreatic cancer cell lines have shown that the thermal sensitization of gemcitabine is best when HT is performed 24 h before gemcitabine (169). Clinical results of randomized trials of HT combined with various CT agents in different tumor sites and histologies have reported positive outcomes (83, 170–172).

In addition, HT-triggered targeted drug delivery systems can ensure an even more effective drug delivery (173–176). Liposomal encapsulation of CT agents increases the half-life of its circulation by avoiding rapid metabolism. Classical PEGylated formulations have been designed to allow passive accumulation of these drugs by exploiting the enhanced permeability and retention (EPR) effect because of the leaky vasculature of the tumors (177). However, such passive drug targeting has been found to achieve insufficient concentration of these formulations within the tumor tissues. This could be improved by temperature triggered release of these thermosensitive liposomes (TSLs) at the tumor site (178). The release of these TSLs could be favorably steered by altering the focus of local heating. Thus, following local HT, TSL drugs are released in the adjacent blood stream, thereby allowing a higher local drug concentration, increasing drug penetration, and improving the bioavailability of the CT agents at the local site. In addition, the TSL loaded with CT agents have several advantages over free drug delivery in terms of treatment monitoring and drug delivery at the local tumor site, thereby minimizing CT-induced normal tissue toxicity. These microcarriers transport the drugs to the target site and even allow image-guided drug delivery (179). The liposomes could pass and concentrate at the tumor site via the EPR effect. Following appropriate external thermal stimuli [loco-regional HT with microwaves (MVs)/radio frequency (RF) waves or high-intensity focused ultrasound (HIFU)], the liposomes release the payload of CT drugs at the desired tumor location.

Presently PEGylated TSL doxorubicin is commercially available and is being evaluated in clinical trials (173, 179–181). Other CT agents, such as payloads are also being actively tested and are undergoing active considerations for introduction in clinics. Some of these include cisplatin, BLM, paclitaxel, docetaxel, and 5-FU (182).

The thermal sensitization of CT has also been used as hyperthermic chemoperfusion in various body cavities, namely, the peritoneum, pleura, and bladder. Some of the drugs, like MMC, oxaliplatin, cisplatin, doxorubicin, paclitaxel, and carboplatin, exhibit thermal synergism at 42–43°C (183). A detailed discussion on this approach is outside the scope of this review. Interested readers may like to further explore through some recent reviews on this approach (184–194).

## Hyperthermia: An Immunomodulator

Hyperthermia at 39–45°C has been demonstrated to modulate the innate and adaptive immune systems and add to its existing radiosensitizing effects and synergistic action with CT agents in the management of cancer (12, 17, 18, 110, 169, 195–197). HT triggers immune responses through the induction of HSPs, especially HSP70, which is released by the tumor cells (Figure 1). On exposing the tumor cells to a higher temperature, protein aggregation and denaturation induce a stress response in the cell, the so-called unfolded protein response. Consequently, increased levels of HSP70 are induced. Because necrotic cells lose their membrane integrity, HSPs act as danger signals outside the cell. Both HSPs and HSP/tumor antigen (Ag) complexes are released. In addition, HSPs and tumor Ag-containing exosomes are discharged from tumor cells. HSP70 containing exosomes derived from heat stressed tumor cells as well as HSP/tumor Ag complexes activate and attract dendritic cells (DCs). The latter take up tumor Ag, present it with co-stimulation to CD8^+^ T cells, and thereby induce cellular antitumor immunity by priming cytotoxic T lymphocytes (CTLs).

Radiation was formerly regarded as an immunosuppressive agent. However, abscopal effects reported with RT both in preclinical models and in clinical situations indicate that the therapeutic effects of RT are partially immune mediated (198–204). It has become clear that RT can provoke both immune suppression and immune stimulation (205). Antitumor CD8^+^ T cells have been shown to be key players in RT-induced immunity (206). The process could be conceptualized sequentially as priming of the tumor Ag specific T cells, migration to and infiltration of the leukocytes in the tumor tissue, and changes in the immunosuppressive tumor microenvironment followed by immunogenic modulation of tumor cell phenotype (204).

**Figure 2:**
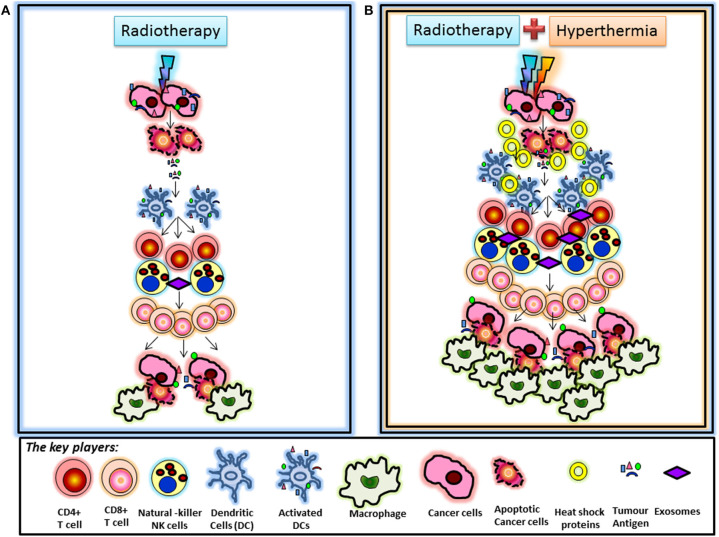
**(A)** Radiotherapy-induced immunomodulation: Immunomodulation induced by radiotherapy is known to be mediated via the sequence of events initiated by activation of dendritic cells, which take up tumor antigens released from cancer cells following radiation-induced cell death. Activated dendritic cells activate T cells leading to a cascade of events resulting in stimulation of cytotoxic CD8^+^ T cells. These cells are further promoted by radiation-induced chemokines to kill tumor cells that are finally scavenged by macrophages. **(B)** Immunomodulation following hyperthermia and radiotherapy: The differential observation of increased CD68^+^ macrophage infiltration in the part treated by combined hyperthermia + radiotherapy could be the result of accelerated immunomodulation secondary to hyperthermia. Hyperthermia along with radiotherapy is known to release heat shock proteins by tumor cells, which may act as additional “danger signals” that may further promote immune responses. Heat shock proteins and antigen-containing exosomes could contribute to dendritic cell recruitment leading to enhanced immunomodulatory effects in this part. This may speed up radiation-induced immunomodulation, as suggested by increased CD68^+^ macrophage infiltration in this part. These interpretations are based on the observation that the tumor specimen taken at 6 weeks showed CD4^+^ T cell infiltration in the part treated by radiotherapy alone, likely representing a first set of events of radiation-induced immunomodulation. In turn, a higher number of CD68^+^ macrophages were found in the part treated by thermoradiotherapy (207).

HT added to RT could thus be expected to enhance the process of immunomodulation. This could be visualized in a reported case of a soft tissue sarcoma where, owing to technical limitations of the tumor size, half of the tumor was treated preoperatively with RT alone whereas the other half received HT in addition to RT (207). The part treated with HTRT showed an enhanced tumor regression as compared with the part receiving RT alone. Further, in the resected tumor specimen at 6 weeks following RT/HTRT, significantly higher number of CD4^+^ cells were evident in the RT-treated part whereas significantly higher number of macrophages (CD68^+^) were observed in the part treated with HTRT. These findings on immunohistochemistry of the resected tumor specimen are a snapshot of the immune events in the parts receiving RT or HTRT and indicate the distribution of the immune cell components after 6 weeks of treatment. Considering the sequence of events that trigger downstream immunostimulatory pathways following RT or HT, one could view the presence of CD4^+^ cells in the RT part and CD68^+^ cells in HTRT part as a reflection of the two separate time points in the sequence of immunomodulatory events stimulated by RT and HT. A higher CD4^+^ could be expected to be an earlier event in the sequence of immune process, whereas a preponderance of CD68^+^ could be a late event reflecting the culmination of the immune process and scavenging the dead tumor cells by CD68^+^ macrophages. Thus, HT in clinical situations appears to be accelerating the entire process of immunomodulation of RT (Figure 2).

In a recently reported phase III randomized study of chemoradiotherapy with HT (HTCTRT) vs. chemoradiotherapy (CTRT) alone in patients with locally advanced cervical cancer (LACC), the authors reported that abscopal effects were significantly higher in patients receiving HT (208). All 210 randomized patients received pelvic RT and two cycles of CDDP. Of these, 108 with extrapelvic disease who survived for a minimum of 6 months were evaluated using ^18^F-FDG PET/CT scans for complete metabolic response at all sites—both within and beyond the pelvic treatment portal (54 patients in each group). At a mean time interval of 196 days (mean: 162–266 days) between end of treatment and evaluation by PET/CT, a significantly higher proportion of patients who received HTCTRT achieved complete metabolic response (HTCTRT vs. CTRT: 24 vs. 5.6%, *p* = 0.013). Even though CDDP was received similarly by both groups of patients, the significantly higher proportion of complete metabolic response observed in both extrapelvic and local diseases in those patients receiving HTCTRT lends support to HT-induced immunomodulation. Thus, along with local immunostimulatory effects of HT to RT as described earlier, the higher abscopal effects with HT suggest even a systemic immune-mediated response resulting in complete regression at sites not within the radiation treatment portal. This effect might even be further enhanced by adding immunomodulators and should be a subject of future clinical trials.

The immunomodulation by HT and its positive impact with RT could introduce a new paradigm that goes beyond the radiosensitizing effects of HTRT combinations. This may further be amplified with the use of immunotherapy with HT/HTRT in various solid tumors (110). However, the optimal temperature for immunomodulation, duration of heating, RT dose and fraction, and the sequence of HT with RT and/or ICIs need further investigation (17, 111).

As intensively having been investigated for RT (205), the immunomodulatory effects of HT can thus be both immune stimulating and immune suppressive (111). During the last decade, the knowledge about the impact of HT on the innate and adaptive immune systems has continuously increased. Several reviews have already been dealing with this topic and are referred to for deeper understanding of the multiple immune functions of HT alone and in combination with RT and/or CT (3, 164, 196, 209).

## Hyperthermia: Swot Analysis

**Figure 3:**
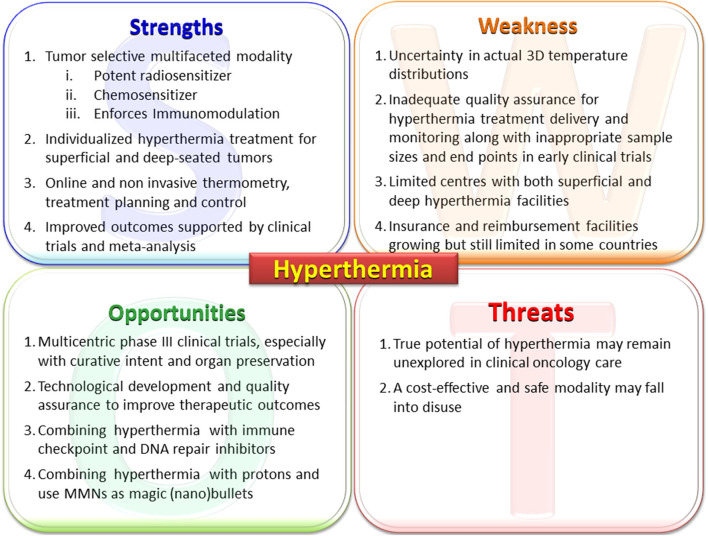
Primary elements of the SWOT analysis for hyperthermia. MMNs, multifunctional nanoparticles; PARP1, poly (ADP-ribose) polymerase-1; DNA-PKcs, DNA-dependent protein kinase catalytic subunit; HSP, heat shock proteins.

The various elements of the strength, weakness, opportunities, and threats of loco-regional moderate HT are discussed below in the form of a SWOT analysis (Figure 3).

## Hyperthermia: Strengths

### Strengths: A Potent Radiosensitizer, a Chemosensitizer, and an Immunomodulator

Moderate HT is a unique therapeutic modality in oncology, with multiple modes of action (Figure 1). Physiological vasodilation following HT improves tumor perfusion and oxygenation of hypoxic cells in particular, rendering them radiosensitive (122, 126, 151). Furthermore, the morphological differences between normal and tumor vasculature (coarse, elongated, dilated, and tortuous capillary network with redundant bending) allow tumors to retain heat through a “heat-trap” effect while heat is washed out from the normal tissues through vasodilation, a “heat-sink” effect (121, 210). This differential heat dissipation provides a selective natural protection for the normal structures from HT-induced radiosensitization. Thus, HT-induced radiosensitization, hypoxic cytotoxicity in deprived microenvironments, and inhibition of DNA damage repair are radiobiologically analogous to attributes of high linear energy transfer (LET) radiation. Hence, HT confers high LET properties to low LET radiations (146, 211).

**Figure 4:**
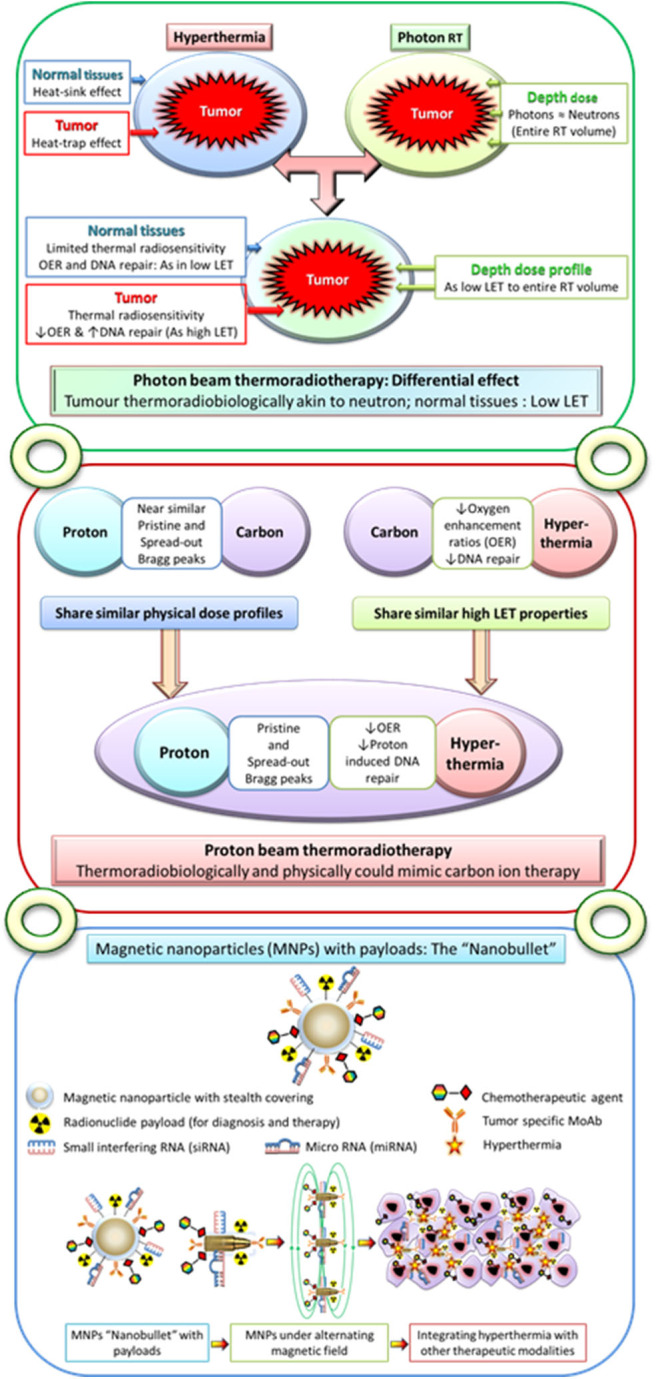
**Upper panel**: Moderate hyperthermia, with its ability to inhibit radiotherapy-induced DNA repair and its radiosensitizing effects on hypoxic tumor cells, has features akin to high linear energy transfer (LET) radiations. Following hyperthermia, the normal tissues exhibit a “heat-sink” effect resulting in the washing off the delivered heat due to heat-induced vasodilation and thus spared from thermal radiosensitization. On the other hand, tumors with its altered vasculature would fail to vasodilate and thus retain heat leading to a “heat-trap” effect. As the physical dose profiles of photons/X-rays are similar to those of neutrons in the irradiated volume, thermoradiobiologically photon beam thermoradiotherapy would have differential effects on tumors and normal tissues. This would be analogous to high LET neutrons for tumor owing to their selective thermal radiosensitization, whereas the normothermic normal tissues would be irradiated with low LET photons/X-rays. **Middle panel**: Protons radiobiologically have low LET radiation features but share similar physical dose profiles with those of high LET ^12^C ions. Thus, hyperthermia with its thermoradiological similarities to high LET radiations when delivered along with protons could mimic C^12^ ion therapy. **Lower panel**: Conceptual illustration of multifunctional magnetic nanoparticles with appropriate payloads of radioisotopes for radiotheranostics (both tumor imaging and radiotherapy) and chemotherapeutic and immunotherapeutic agents and siRNA and miRNA for therapeutic gene silencing. These magnetic nanoparticles in the presence of alternating magnetic field could deliver local hyperthermia and with additional payloads could act as “nanobullets.” The figure has been reproduced and modified with permission from Datta et al. (212, 213).

Photons have depth dose profiles similar to those of neutrons but exhibit low LET features. Normal tissues irradiated with low LET photons are spared from thermal radiosensitization through “heat-sink” effect (212) (Figure 4). However, the “heat-trap” phenomenon results in selective thermoradiosensitization of tumors, enabling the low LET photons to acquire high LET features similar to those of neutrons. Photon thermoradiotherapy (HTRT) could therefore result in improved therapeutic outcomes without additional morbidity. This is confirmed by results from most photon HTRT studies (12, 21–68, 84–93, 95).

As with RT, the thermal sensitization of many CT agents is mediated through their interference with the DNA repair (106). HT with CT inflicts a wide range of DNA damage including strand cross-links, and single-strand and double-strand DNA breaks. The repair of these damages could involve excision repair, homologous recombination, standard NHEJ, and alt-NHEJ. HT is known to inhibit these repair pathways, although its influence on inhibiting NHEJ repair is debated, especially on the alternative NHEJ repair pathways. The inhibitory effect on HT on CT-induced DNA damage could get further amplified when RT is used concurrently with CT (214). Thus, a trimodality approach, using HT along with RT/CT/CTRT, has been reported to result in favorable outcomes in various malignancies (69–83, 91, 94). Further clinical studies need to be undertaken to optimize the sequence of these modalities and the appropriate CT agents for specific neoplastic conditions.

As discussed above, recent *in vitro* and *in vivo* studies suggest that HT can reinforce immunomodulation when combined with RT through a chain of events (111). *In vitro* and *in vivo* studies using even radioresistant melanoma cell lines have shown an increased release of HSP70 and high mobility group box protein 1 (HMGB1) with HT and RT. *In vivo*, significantly higher amounts of Ag-presenting DCs were only found in tumors that were treated with HT and RT (16). Such immune alterations can trigger both local and systemic antitumor reactions (198, 215, 216). Thus, HT at 39–45°C could elicit antitumor immune response by a multitude of mechanisms, and distinct enhanced temperatures have distinct immune-modulating properties that ideally do complement each other. These include enhancing the surface expression of MHC class I-related protein A (MICA), HSPs, and/or exosomes; activating NK cells, CD8^+^ T cells, and DCs; and augmenting the immune-cell trafficking between the tumor and lymphoid organs (197). These all could contribute toward both local and systemic immunostimulation by HT. Further, with the increased use of immunotherapy with RT in various disease sites, addition of HT could be expected to further amplify the therapeutic outcomes (110, 204, 217–219). Thus, HT in multimodal tumor therapy settings can be considered as mirroring “*in situ* tumor vaccination” in cancer and hence holds a great promise in getting integrated in future therapeutic strategies in clinics (19, 20, 111, 220–223).

HT is thus a unique and versatile therapeutic modality capable of favorable interactions with RT and/or CT and is a potential immunomodulator. These multifaceted attributes could be taken into advantage by integrating HT with other modalities in routine clinical management.

### Strengths: Individualized Hyperthermia for Superficial and Deep-Seated Tumors

Individualized HT delivery is important to achieve good clinical results, and therefore, the heating equipment should be tailored to the individual patient's requirements. Most clinical HT devices use RF, MW, electromagnetic radiation, ultrasound, or infrared (IR) light sources (2, 224–230). MW applicators (434–915 MHz) and IR (>300 GHz) are used to apply local HT to superficial tumors, which infiltrate ≤4 cm below the skin (224). The first-generation clinical MW applicators achieved limited heating of only 30–60% of the aperture face and was only effective for small lesions (231). Technical developments have resulted in modern equipment capable of treating larger tumor areas. Furthermore, dedicated intracavitary and phased-array MW devices have been developed for treating, for example, bladder cancer (172, 225) and head and neck tumors (226). Presently, good-quality superficial MW systems are capable of adapting to various tumor sizes and shapes, by using arrays of antennae or differently sized (body-conformal) antennae (225–228). IR can effectively heat very superficial (<2-cm depth) widespread local diseases (37, 229).

Loco-regional HT is applied to more deep-seated tumors. Early HT trials conducted with first-generation devices were unable to steer or focus energy other than by shifting the patient with respect to the applicator. High-quality heating of the tumor, without overheating normal tissues outside the target volume (hot spots), was challenging with such devices (171, 232, 233). Modern phased-array systems with 4–12 antennas or antenna pairs organized in one to three rings around the patient (234, 235) operate at 60–150 MHz to achieve a spatial control of ~5 cm over the power distribution. These individually controlled RF antennas allow much better focusing of heat onto the target volume and adequate control to avoid hot spots (104). They allow safe and good-quality individualized HT treatments for both superficial and deep-seated tumors. A novel approach is to induce localized moderate HT of deep-seated tumors by scanning a HIFU beam through the tumor volume. This is currently under development with the specific intent to use controlled moderate HT in the tumor with temperature-sensitive liposomal drugs to achieve tumor-specific drug delivery (236–238). A proof-of-concept study has demonstrated that this HIFU-based moderate HT method can result in superior tumor temperature uniformity, when combined with sophisticated fast online non-invasive temperature feedback data by non-invasive MR thermometry to maintain stable temperatures (239).

### Strengths: Online and Non-invasive Thermometry, Treatment Planning, and Control

Achieving adequate thermal dose is crucial owing to the strong dose–effect relationship (163, 240). However, obtaining optimal individual applicator settings that yield high tumor temperatures without overheating the normal tissues is complex with multiple independent antennae and requires optimization and phase-amplitude steering during treatment. Real-time HT should be monitored using minimally invasive thermometry probes in or near the tumor. Non-invasive MR-based 3-D temperature maps and treatment planning are now available. Non-invasive MR thermometry is now widely used for MR-guided HIFU-based thermal ablation procedures (241). The proton resonance frequency shift (PRFS) method measures relative temperature changes with a resolution of ±1°C as validated for soft tissue sarcoma in the pelvis and lower extremities (105, 242); non-invasive MR thermometry is increasingly used for monitoring moderate HT treatment, presently mainly at tumor sites with minimal motion artifacts (e.g., soft tissue sarcoma in extremities), as accuracy of PRFS can be lower in body regions with significant respiration, cardiac, or bowel organ motion artifacts (243–245). Model-based and other new MR thermometry temperature reconstruction methods are emerging. These are capable of ensuring acceptable temperature resolution in the presence of motion artifacts, which will render MR thermometry feasible for many deep-seated tumor sites (241, 246–248). A positive correlation was found between tumor temperature levels obtained by MR thermometry and pathologic tumor response for soft tissue sarcoma (105, 249).

**Figure 5:**
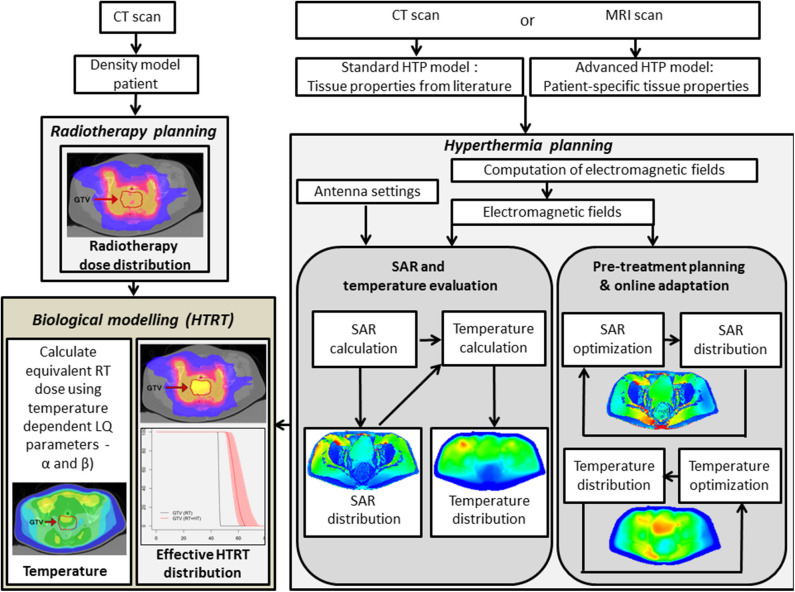
Flowchart depicting the hyperthermia treatment planning process, including SAR and temperature optimization, delivery, and online adaptive optimization. In addition, the hyperthermia and radiation therapy treatment plans are integrated in a joint platform using biological modeling with temperature-dependent linear-quadratic parameters α and β. RT, radiotherapy; HTP, hyperthermia treatment planning; HTRT, thermoradiotherapy; SAR, specific absorption rate. The images of HTP, RT, and HTRT plans have been modified and reproduced with permissions from Kok et al. (261) and van Leeuwen et al. (262).

HT treatment planning simulates 3-D power and temperature distributions. Inverse planning algorithms for optimizing antenna settings are comparable with the planning standards in RT (250–252). Inverse HT treatment planning has been in use in large patient series and found to be qualitatively reliable in predicting initial treatment settings (253–255). In the absence of patient-specific tissue properties and perfusion values, data extracted from the literature display a large range of values, rendering treatment planning quantitatively unreliable (256–258). A correction on these initial settings is possible as a correlation exists between measured and simulated changes in absorbed power and temperature resulting from phase and amplitude steering during HT treatments (259). Thus, while an HT session commences using the settings prescribed by inverse planning, during treatment, online invasive or non-invasive MR thermometry temperature data feedback can assist in adaptive phase-amplitude steering to optimize treatment delivery (96, 99, 259, 260). An example of the work flow is depicted in Figure 5.

**Figure 6:**
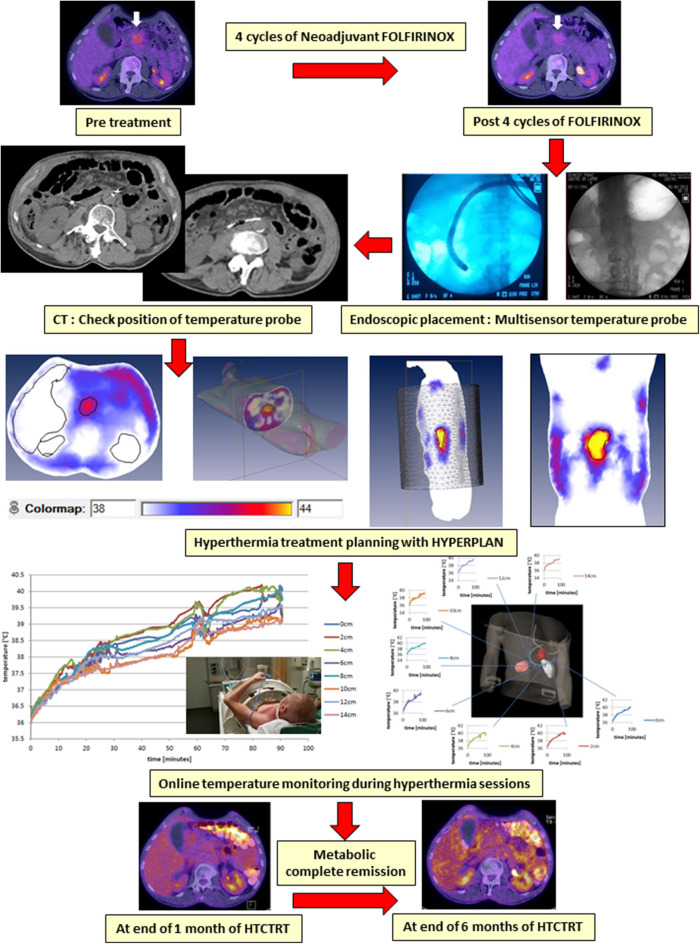
An illustrative example of a patient with locally advanced pancreatic cancer being treated with hyperthermia and radiotherapy as per the HEATPAC protocol (264). Multisensor thermometry probes are guided through the endoscope and placed in the duodenum. Hyperthermia treatment plan is obtained using HYPERPLAN, and the online temperature monitoring is carried out during the hyperthermia. The tumor shows metabolic complete remission on PET obtained at 6 months following the treatment with hyperthermia, radiotherapy, and chemotherapy (HTCTRT).

In cases where invasive temperature monitoring within the tumor may not be practically feasible, one could look for placing thermometry probes in proximity to the tumor. This minimally invasive approach is routinely used for tumors in proximity to natural cavities (the cervix, bladder, rectum, and pancreas) and is justified based on the large and fairly uniformly heated target region during loco-regional HT (263). This approach is illustrated in a patient with locally advanced pancreatic cancer undergoing HT, treated as per the HEATPAC protocol (264). The multisensor probes are placed endoscopically in the duodenum so as to lie adjacent to the pancreatic tumor mass. This permits real-time temperature monitoring during each HT session and also avoids the untoward risk of invasive thermometry placement and retention within the tumor for the total duration of 6 weeks of treatment (Figure 6). Although the thermometry provides an indicative temperature in close proximity to the primary tumor, this should be carried out only in circumstances where invasive thermometry could jeopardize patient's safety and tolerance. In all other situations, attempts should be made for direct invasive tumor temperature monitoring, if possible supplemented with non-invasive MR thermometry, if such facilities are available (104).

Online thermometry with non-invasive MR thermometry and/or treatment planning also enables operators to modify the system and antenna settings to optimize the spatial thermal dose distribution within the tumor. Online re-optimization of antenna settings, aided by temperature feedback and online adaptive planning, has improved treatment delivery (96, 99, 260, 265). Presently, MR thermometry is very helpful to map online the 3-D temperature distribution and assist in adjusting treatment settings.

### Strengths: Improved Clinical Outcomes With Hyperthermia—Clinical Trials and Meta-Analysis

**Figure 7:**
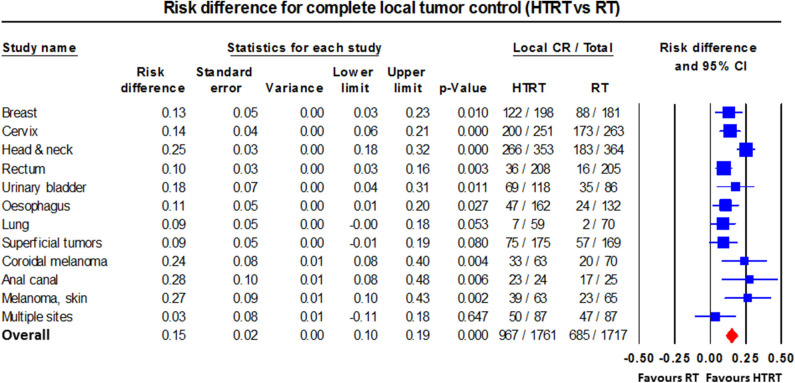
Forest plot showing the risk difference of complete local tumor control in patients from various disease sites treated with thermoradiotherapy (HTRT) vs. radiotherapy (RT) alone. Plot generated using data from Datta et al. (12).

Single-arm and randomized clinical trials of RT/CT/CTRT with/without HT have mostly reported favorable outcomes in a range of primary and recurrent cancers. These include various superficial tumors (22, 23); melanoma (24); choroidal melanoma (66); brain tumors (61, 62); malignant germ cell tumors (82); soft tissue sarcoma (83, 266); bone metastases (59, 60); LACC (41–47, 63, 69, 74, 76, 91–93); locally advanced head and neck cancer (LAHNC) (26–33, 71, 79, 88); cancers of the esophagus (80, 81, 94), breast (34–40, 89, 90), lung (67, 68), pancreas (72, 264), urinary bladder (55–58, 70, 75), prostate (49–53), rectum (64, 65, 73, 95), anus (77, 78), and pelvis (54); and other tumors (12, 21, 84, 85, 87). An overall CR of 54.9% with HTRT vs. 39.8% with RT alone (risk difference = 0.15, 95% CI: 0.11–0.20, *p* < 0.001) was reported from 38 clinical trials of HTRT vs. RT alone in 3,478 patients with various tumor sites (RT, *n* = 1,717; HTRT, *n* = 1,761) (12) (Figure 7).

Local HT seems unlikely to significantly improve survival outcomes in palliative indications (e.g., recurrent breast cancers and those with metastatic disease), although it can provide effective palliation (22, 24, 89, 90, 267). Nevertheless, significant long-term survival benefit (*p* = 0.03) at 12 years along with local control (*p* = 0.01) was reported in newly diagnosed LACC treated with HTRT vs. RT alone (43). The phase III randomized trial (EORTC 62961-ESHO 95) in high-risk soft tissue sarcoma also reported significantly improved local progression-free survival (*p* = 0.02) and overall survival at 10 years with neoadjuvant HTCT over CT alone (*p* = 0.04) (83).

Loco-regional HT also permits de-escalation strategies, maintaining tumor control while reducing side effects. The randomized RADCHOC trial found HTRT yields similar results as CTRT, without the CT associated side effects in LACC patients (45). As HTRT further reduces the tumor α/β, the outcomes could be further improved by hypofractionation, thus even allowing for RT dose de-escalation.

Human papillomavirus (HPV) is a known etiopathological agent for cervical cancer. In HPV-positive cervical cancer cell lines, HT activates p53-dependent apoptotic pathways (268). Further using serial quantification of HPV titers from the local cervical cancer in LACC treated with RT alone, a serial reduction in HPV titers with cumulative RT doses correlated with increasing local tumor control (269, 270). This has also been proposed to be through the restoration of apoptotic pathways in HPV-positive tumors. Thus, a combination of HT and RT in HPV-positive cervical cancer, both of which restore the apoptotic pathways in HPV-positive cervical cancer, could form a viable alternative to CTRT. This approach could logically be extended to HPV-positive LAHNC in which HPV-positive tumors could be treated with HTRT alone. A stratified randomized study based on HPV status in LACC and LAHNC is warranted to examine this likely advantage with HTRT in HPV-positive tumors.

Systematic reviews and meta-analyses provide level I clinical evidence of a therapeutic approach. The International Collaborative Hyperthermia Group meta-analysis reported an overall CR of 59% with HTRT vs. 41% with RT [odds ratio (OR) = 2.3, 95% CI: 1.4–3.8, *p* < 0.001] in superficial localized breast cancer (89). A meta-analysis in recurrent breast cancer reported CR of 60.2% with HTRT vs. 38.1% for RT (OR = 2.64, 95% CI: 1.66–4.18, *p* < 0.0001) (90). Of the 779 patients reirradiated with an additional mean dose of 36.7 Gy with HT, 66.6% achieved CR (event rate = 0.64, 95% CI: 0.58–0.70). In LAHNC, HTRT improved CR vs. RT alone (62.5 vs. 39.6%; OR = 2.92, 95% CI: 1.58–5.42, *p* = 0.001) (88). A Cochrane Review in locally advanced rectal cancer reported a better 2-years overall survival with HTRT compared with RT (hazard ratio = 2.06, 95% CI: 1.33–31.7, *p* = 0.001) (95). In advanced esophageal cancers, the overall survival at 1–7 years was significantly higher with thermochemoradiotherapy (HTCTRT) over CTRT (94). The Cochrane meta-analysis conducted in 2010 in LACC reported improved local tumor control (hazard ratio = 0.48, 95% CI: 0.37–0.63, *p* < 0.001) and overall survival (hazard ratio = 0.67, 95% CI: 0.45–0.99, *p* = 0.05) with HTRT vs. RT without any increased acute or late morbidity (93). A recently updated meta-analysis has confirmed the above results (92). Thus, level I evidence of a therapeutic advantage with HT added to RT and/or CT has been evident in various sites.

Network meta-analysis (NMA) is considered the highest level of clinical evidence and a vital tool for treatment guidelines and decision making by various national and international organizations including the World Health Organization (WHO). NMA helps to identify the best therapeutic option and provides level I clinical evidence in situations with multiple treatment options. An NMA in LACC evaluated 9,894 patients from 59 randomized trials treated with 13 different therapeutic options (91). Taking the cumulative effects of all the key primary endpoints into consideration, the results ranked HTRT and HTCTRT as the top two options in LACC. These results of NMA favoring HT in LACC over all other approaches are noteworthy (271, 272).

Adding HT to RT and/or CT has not been shown to increase the acute or late morbidity. In a recently reported pilot study of RT and local HT in 16 elderly patients (median age: 81 years), all patients had a CR with functional bladder preserved following treatment, but none of them had any grade III/IV acute or late genitourinary and gastrointestinal toxicities (56). The meta-analysis in LACC comparing patients treated with HTRT vs. RT reported that there was no significant increase in grade III/IV acute morbidity (risk difference: 0.015, *p*: not significant) and grade III/IV late morbidity (risk difference: 0.039, *p*: not significant) (92). In 19 randomized control trials that included 1,519 patients with locally advanced esophageal cancers, adding HT to CTRT or to RT did not show any significant increase in acute hematological, esophageal, or pulmonary toxicity (94). Even when HT is added with RT for reirradiation in recurrent breast cancers, patients have not experienced late morbidity (267). These reports from various tumor sites indicate that HT when added to RT and/or CT is both safe and efficacious.

## Hyperthermia: Weakness

### Weakness: Uncertainty in Actual 3-D Temperature Distribution

Temperature registered by thermometry probes during HT represents only a small sub-volume of the target region. However, reported dose–effect relationships confirm that minimally invasive thermometry is indicative of thermal dose for pelvic tumors (163). For superficial HT, treatment quality can be guaranteed by superficial probes (>50 stationary sensors/400-cm^2^ applicator aperture area) with one or more invasive probes (273). Thus, the available thermometry probes allow adequate treatment guidance, and therefore, the absence of fully accurate 3-D temperature information is not a fundamental weakness and likely to improve with the ongoing developments in MR thermometry and HT treatment planning (96, 260). Hybrid MR-guided HT devices with MR thermometry are now in routine use in several centers, and these could provide a real-time estimate of the temperature profile in the tumor volume and also adjacent normal structures (105, 242).

### Weakness: Inadequate Quality Assurance for Hyperthermia in Early Clinical Trials

Adequate quality assurance is of utmost importance in clinical practice. However, especially earlier studies sometimes lacked adequate sample size, strict patient selection criteria, suitable endpoints, strict adherence to treatment protocols, or quality assurance. Even some trials conducted by major collaborative groups suffered from these weaknesses (23, 171, 231, 274, 275). Clinicians might overlook these deficiencies in heating technology and temperature monitoring and inadvertently interpret negative results from weak studies as demonstrating a lack of benefit from HT. The resulting negative impression of HT might have significantly hindered the acceptance of HT in routine clinical practice. The RTOG 89-08 study, which followed the earlier RTOG 84-01 (23), was carried out using a second-generation HT system capable of steering the heat deposition (276). Although 74% of the patients included in the RTOG 89-08 study had recurrent or persistent tumors, 34% achieved CR. In those receiving ≥45 Gy of RT, the CR was 54% compared with 7% with <45-Gy RT along with HT (*p* < 0.0001). Moreover, the compliance with HTRT improved to 68% compared to just 18% with the previous equipment used in RT 84-01 study (23). Thus, it is mandatory to ensure the use of modern equipment, quality assurance, and temperature monitoring in all future HT studies to be able to assess the true therapeutic potential of HT. These requirements are also highlighted in the recently published quality assurance guidelines from ESHO (2, 224, 277). Strict compliance to these guidelines is therefore desirable and should be adopted in all future clinical trials with a HT.

The impact of adequate thermal dose has been demonstrated in many studies (240, 278) and has recently been confirmed as an independent prognostic predictor of local control and disease-specific survival in 227 patients with LACC, treated with state-of-the-art RT and HT (163). This further reinforces the value of quality assured HT along with optimal RT to achieve optimum results with HTRT. Modern equipment complemented by HT planning, online thermometry, MR-guided HT, and strict adherence to quality assurance play a pivotal role to enhance therapeutic outcomes with HT.

### Weakness: Limited Centers With Superficial and/or Deep Hyperthermia Facilities

Presently, HT facilities are restricted to only a few centers. The majority have superficial HT systems, whereas some also have deep HT units. The devices use either electromagnetic (radiative/capacitive), ultrasound, or IR heating technologies (2). A list of HT centers globally is not available in the public domain. According to the ESHO website (279), only 20 centers in six countries employ HT for cancer therapy. In comparison, there are in excess of 1,500 RT centers in Europe (280). The development of more HT centers requires education about HT and its quality assurance through training programs endorsed by national and international radiation oncology societies. A larger number of centers with superficial and deep HT facilities would facilitate recruitment and timely completion of well-powered randomized trials in various disease sites with HTRT and/or CT.

### Weakness: Insurance and Reimbursement Facilities

Within Europe, reimbursements for HT are variable. Both superficial and deep HT are reimbursed regardless of indication in Italy, Poland, and the Czech Republic, whereas in Germany, the Netherlands, and Switzerland, reimbursement is restricted to indications supported by the strongest clinical evidence (281, 282). The USA reimburses palliative HT treatment for superficial tumors and deep HT for only those patients with LACC who cannot tolerate CT. Information regarding reimbursement in countries outside Europe is limited, but HT is reimbursed in some major Asian countries. Reimbursement is slowly growing and will benefit from the outcomes of phase III trials in other tumor sites.

Presently, the evidence from phase III trials supported by pairwise and/or NMA is limited to a few sites like LACC, LAHNC, esophageal cancers, and loco-regional recurrent breast cancers (88–94). This underlines the need for randomized phase III trials in other sites especially with curative intent and aiming at organ preservation. This would pave the way for including such sites with positive outcomes in the list of reimbursement. Furthermore, it is also important to apprise the decision-making bodies with the recent developments in HT. This has been effective in Switzerland where the federal authorities have agreed to reimburse HT for a number of clearly well-defined indications, for both superficial and deep HT (282).

## Hyperthermia: Opportunities

### Opportunities: Multicenter Phase III Randomized Clinical Trials

Current evidence from several randomized clinical trials and meta-analyses should encourage a larger number of well-designed phase III randomized clinical trials with adequate sample sizes and proper patient selection to evaluate logical endpoints. These should be performed primarily in the curative setting, and especially in patient groups where the outcome with conventional approaches remain unsatisfactory and with emphasis on exploiting the tumor-selective radiosensitization to gain clinical benefit in key survival endpoints, functional and organ preservation, quality of life, compliance, toxicity profiles, and cost efficacy.

In view of the limited number of HT centers, it is imperative for all centers to participate in multicenter clinical trials under the auspices of national, regional, or international HT scientific societies and strictly follow quality assurance guidelines for heating and temperature monitoring.

### Opportunities: Technological Development and Quality Assurance

The recent advances in system design provide adequate treatment control to ensure effective heating. Quality assurance, non-invasive thermometry, and treatment planning offer an opportunity to further improve treatment delivery in terms of tumor temperature achieved, optimization of synergy with concurrent RT and CT, and ease of operation. Moreover, techniques for highly targeted heating are developed, such as mild HT by ultrasound triggering the local delivery of CT and thereby providing targeted chemoablative response (181).

Treatment guidance, based on online temperature feedback combined with information from MR thermometry data or treatment planning predictions, has shown to improve tumor heating and reduce treatment-limiting hot spots (96, 265). MR-based treatment guidance is standard in thermal ablation and increasingly applied in mild HT of large fixated tumors and as part of ongoing research (105, 242, 283). Current research focuses also on the improvement of the quantitative reliability of (inverse) treatment planning by reconstruction of patient-specific tissue characteristics using MR-electrical properties tomography (284, 285) as well as incorporating the thermal impact of heat flow in fully realistic vessel trees (100, 286–288). User-friendly software integrated in an intelligent user-interface provides robust optimization algorithms based on temperature and hot spot feedback during treatment. These could further improve treatment quality and reduce operator dependency of treatment guidance.

Biological modeling using temperature-dependent values of the LQ parameters α and β provides a unique method to quantify the clinical effect of HT in terms of an equivalent enhanced radiation dose (289, 290). These models could answer highly relevant clinical research questions on the basis of optimally exploiting the synergy between HT and RT, similar to biological modeling for RT (291–293). An example is the evaluation of the effect of the time interval between RT and HT showing that radiosensitization increases significantly and tumor selectively for shorter time intervals (102). Further, *in vivo* and clinical data have shown that shorter time intervals are more effective (142, 148). Simulations have also shown that the additional effect of HT to RT total dose in realistic clinical scenarios is typically in the order of 10 Gy without inducing more side effects, which conforms to the reported clinical benefits of this combination (289, 290, 294).

Future research will focus on further modeling the environment to predict and optimize the biologically equivalent HTRT dose to improve therapeutic outcomes. Mature HTRT biological planning will ultimately allow full and robust exploitation of the differing physical and biological properties of RT and HT to achieve a high tumor control with acceptable side effects.

### Opportunities: Hyperthermia With Immune Checkpoint and DNA Damage Repair Inhibitors

ICIs have yielded promising results in certain tumors (222, 223). However, presently only less than half of the tumor types are eligible for ICIs (295). The low efficacy of ICIs could be due to inadequate delivery of these agents to the local tumor site and reduced accessibility of tumor neo-antigens to the antigen-presenting cells (APCs) (19, 20, 296). HT could improve the delivery of ICIs to the tumor sites through vasodilation. Furthermore, through release of HSPs and activation of the recruitment of tumor killing immune cells, HT can itself initiate the chain of events toward enhancing the antitumor immune response (16–18). HT also accelerates RT-induced immunomodulation (16, 207). Increasing the efficacy of both ICIs and RT by HT could create novel therapeutic opportunities and could be explored in future randomized trials especially in malignant melanoma, head and neck cancers, triple negative breast cancers, cervical cancer, and bladder tumors (19, 20, 110, 111, 297).

The inhibition of HR repair for DNA DSBs is a key to the radiosensitizing action of HT (106, 139). In addition to HR, the c-NHEJ and alt-NHEJ have been also associated with repair of DSBs (133). HT could also partly affect these pathways (137, 298), but specific agents have been shown to be even more effective in targeting key proteins in the c-NHEJ and alt-NHEJ pathways. DNA-dependent protein kinase catalytic subunit (DNA-PKcs) and poly (ADP-ribose) polymerase 1 (PARP1) play key roles in the c-NHEJ and alt-NHEJ repair pathways of DNA DSBs, respectively.

In addition, HSP90 induced by HT inhibits HR repair (133). Combining HT with inhibitors targeting the above proteins could lead to a comprehensive reduction in DNA repair, thereby increasing cell kill and effectively leading to “synthetic lethality” (108). *In vitro* studies have shown a favorable interaction of HT with such DNA damage response inhibitors resulting in amplification of the tumor cell kill (133, 136–138). It may therefore be of interest to evaluate these agents in conjunction with HT in future clinical trials.

BRCA2 is a pivotal protein for HR repair. HT can temporarily downgrade BRCA2 and inhibit HR, even in BRCA2-proficient cells (109). This induces transient synthetic lethality by HT in these cells. The addition of cytotoxic agents, such as CDDP to HT could further increase tumor cell kill. *In vitro* experiments have confirmed that HT, along with a PARP1 inhibitor and CDDP, could boost synthetic lethality even in BRCA2-proficient tumor cells (107–109, 134, 135). These results open up possibilities for translating novel therapeutic strategies with HT aiming at “synthetic lethality.” Clinical feasibility of these strategies is currently being explored in feasibility studies.

### Opportunities: Hyperthermia With Protons and Use as Multifunctional Magnetic Nanoparticles

Until now, HTRT has been used primarily with photons. However, with the recent increase in the number of proton centers, it would be appropriate to investigate the potential benefits of proton HTRT. Like photons, protons are low LET radiation, but physically, their dose profile with a Bragg peak is similar to that of ^12^C ions. Protons supplemented with the high LET features of HT could mimic ^12^C ion therapy and thus be a cost-effective alternative to ^12^C ions (299–303) (Figure 4). Proton HTRT has shown promising results in experimental choroidal melanomas and inoperable large sacral chordomas (304, 305). Presently proton HTRT is under investigation in inoperable soft tissue sarcoma (266, 299).

Multifunctional magnetic nanoparticles (MMNs) provide yet another attractive means to integrate HT with diagnostic and therapeutic modalities into a single cancer “theranostic” vector (Figures 1, 4). A leaky tumor vasculature allows the nano-sized particles to passively escape through the tumor endothelial cells to the interstitial fluid. This is feasible as the intercellular gaps in the endothelial cells can reach up to 4 μm compared to <10 nm in normal vasculature. Furthermore, the impaired tumor lymphatic architecture and drainage result in preferential accumulation and retention of these nanoparticles at the local site. MMNs thus extravasate preferentially into the tumor tissue and are retained by capitalizing on the EPR effect (306–308).

Magnetic nanoparticles (MNPs) could be potentially delivered to the tumor via direct infiltration, intraperitoneal, intra-arterial, intracavitary, and intravenous administration (309). An adequate concentration of MNPs in the tumor volume is desirable for adequate heating. Direct tumor infiltration could ensure a higher intratumor concentration of MNPs, but its application would be limited depending on the tumor location. A systemic administration could permit a higher internalization but results in low accumulations due to phagocytosis and elimination by the reticuloendothelial system. Moreover, the availability of MNPs at tumor site would also depend on the EPR effect that allows them to extravasate into the tumor parenchyma. However, the EPR effect could be compromised by the abnormal tumor vasculature, raised tumor interstitial pressure, and the deregulated extracellular matrix. All these could hinder the EPR effect and thus limit the biodistribution of the MNPs (310–312). Currently, various strategies are directed toward either enhancing the EPR effect or bypassing it. The EPR could be augmented through inducing systemic controlled hypertension using angiotensin II along with MNPs that results in improving tumor perfusion (313). Other methods target the tumor microenvironment to promote vascular supply and perfusion using photoimmunotherapy (314) or radiation therapy (315) prior to administration of nanoparticles. These could facilitate migration of MNPs into the tumors by damaging the perivascular cells. Fluorescent peptides have also been investigated to bypass the EPR. Upon entering the tumors, they could be cleaved by the enzymes of the apoptotic cells to form nanoparticles (316) or by encapsulating or tethering microbubbles with the nanoparticles (317). These microbubbles could then induce transient vascular permeability when sonicated by ultrasound owing to cavitation and microstreaming effects.

The application of an alternating magnetic field (AMF) magnetizes these MNPs in opposite directions, resulting in energy deposition through hysteresis losses. Local HT is thereby produced owing to rotation of the nanoparticles (Brownian relaxation) and flipping of the magnetic dipoles owing to rotation of the magnetic moments in the magnetic field (Néel relaxation) (318). This results in heating the tumor from the “inside-out.” On the contrary, conventional hyperthermia techniques (with MWs and RF) achieve an “outside-in” heating of the tumors (319–321) resulting in unavoidable power losses and the undesirable, inevitable concomitant heating of the intervening normal structures.

The ability to achieve adequate heating at the tumor site depends on the local concentration of the MNPs, the applied frequency, and field strength of the AMF. Presently, the only clinically available AMF system, NanoActivator (MagForce AG, Germany) with a diameter of 20 cm, operates at a frequency of 100 kHz and with field strength of 18 kA/m (309, 322, 323). Although the patients with glioblastoma multiforme (GBM) were reported to tolerate the above frequency and field strength, those with prostate cancers could bear around 5 kA/m (324–326). The median maximum temperatures of 44.6°C (42.4–49.5°C) with T90 (the temperature exceeding 90% of the tumor) of 40.5°C could be achieved in GBM (325). In pelvic tumors, 86% of the target could be heated to >40°C, whereas coverage of >42°C was reported as unsatisfactory (327). This was attributed to the smaller radius of the head compared with the pelvis. As higher field strength and frequencies could result in improved heating, efforts are ongoing to improve these parameters and also to develop better skin surface cooling for areas, especially in the pelvis (309). In addition, an optimum temperature needs to be achieved and monitored to improve the tumor control and minimize the damage to adjacent normal tissues. A single-point thermometry may be inadequate, whereas a multipoint thermometry could pose problems with spatial resolution. A temperature feedback control system that could adjust the AMF parameters in real time is therefore desirable to optimize the heating within the target volumes with MNPs (323).

Conjugating tumor-specific peptides and antibodies onto the surface of these nanoparticles aim at improved tumor specification. In the presence of AMFs, MMNs induce local HT and off-load their payloads. The latter can be individually designed and tailor-made with tumor-specific radiotheranostics (for both imaging and RT), CT drugs, immunogenic agents, and gene silencing therapy.

Clinical studies with MNPs in recurrent GBM and recurrent prostate cancers have shown promise, and thus, MMNs are currently under investigation to widen the scope of their clinical application in other malignancies as a potential theranostic agent (324, 325, 328–337). Thus, MNPs could form an alternate mode to integrate hyperthermia along with other therapeutic and diagnostic agents that could be tailor-made to a particular tumor type. This would further potentiate MMNs toward becoming “magic (nano)bullets,” a tumor-specific multimodal therapy with HT (213, 338).

## Hyperthermia: Threats

### Threats: True Potential of Hyperthermia in Various Tumor Sites May Remain Unexplored

The limited availability of HT results in a lack of the awareness of the potential of HT among both clinicians and patients. As discussed, HTRT improves local tumor control in previously untreated LAHNC and LACC by 23–25% over RT alone (88, 92). Moreover, HTRT resulted in a CR in two-thirds of patients with recurrent breast cancers, which is 22% higher compared with that of RT alone (37, 90). Even in previously irradiated patients, HT with moderate doses of RT resulted in a CR of 66.6% (90). These results have led to the adoption of HTRT in the German and Dutch guidelines for recurrent breast cancer (339, 340). The potential benefits of HT in other cancers may remain unexplored for lack of HT facilities.

Scarcity of HT facilities thus will deny patients the established and potential benefits of this multifaceted therapeutic modality across a wide range of tumors. As noted, HT with moderate RT doses can result in very effective palliation in recurrent breast cancer that failed in all other standard therapies (90, 267). In cancer of the anal canal, HT with CTRT has been shown to improve significantly the overall survival and disease-free, local disease-free, and colostomy-free survival rates as compared with CTRT alone (77). A pilot study in elderly patients with muscle invasive bladder cancer unfit for definitive surgery or CTRT showed that following HTRT, a local control of 93.7% with 100% bladder preservation could be achieved without grade 3 or 4 acute or late toxicities (56).

With the gradual aging of the society and increasing cancer incidence, HT with RT could provide a viable and effective option for such elderly patients and increase organ preservation. Furthermore, the tumor-selective thermal radiosensitization could enable RT/CT dose de-escalation to improve patient compliance and avoid toxicities in these vulnerable patients, while maintaining good clinical results.

### Threat: A Cost-Effective and Safe Modality May Fall Into Disuse

HT involves a one-time investment with minimal recurring costs. A loco-regional HT device could be expected to cost around US$ 0.5–1.0 million. However, unlike RT, the daily patient throughput is lower, as each treatment takes around 90–120 min. Thus, in an 8-h working day, four to five patients can be treated/day/unit, that is, 20–25 patients/week, as HT is usually not delivered daily but once or twice a week (43, 77, 88, 92). Thus, 170–325 patients/year could be treated with HT, if delivered once a week for 4–6 weeks. Assuming a working life of 10 years for HT unit, the cost/patient could vary between US$ 154 and 294/patient for a unit costing €0.5 million. This would be US$ 307–588/patient for a capital investment of US$ 1 million investment. For twice-weekly HT, around 85–162 patients could be treated annually with the cost/patient twice for each of the corresponding figures with once-a-week HT. These figures should be regarded as indicative only. A detailed cost analysis is beyond the scope of this manuscript.

Nevertheless, a preliminary cost analysis indicates that setting up HT could be cost-effective and would yield positive returns on investment depending on the patient throughput, user charges, and departmental policies of using 1- or 2-weekly fractions of HT. In the Dutch Deep Hyperthermia Trial in LACC, HT with RT was proven to be cost-effective with maximum discounted cost/life-year gained of about €4,000 (341). In view of the increasing concerns of cancer treatment costs globally, similar cost-effective studies with HT from other countries are highly desirable.

HT is *sui generis* with its multifaceted therapeutic actions—a potent radiosensitizer, a chemo-sensitizer, and an immunomodulator without any significant additional acute or late normal tissue morbidity. Adding HT to other therapeutic modalities could be expected to be safe and cost-effective and thus could be of special value also to countries with limited financial resources.

Combining HT with proton therapy has a thermoradiobiological rationale (299). Early results with proton HTRT in unresectable sacral chordomas and soft tissue sarcomas are promising (266, 305). Confirmation of the hypothesis that proton HTRT is analogous to ^12^C ions could result in major savings over investments in ^12^C ion therapy. The capital cost is estimated at €138.6 million for a combined carbon-proton setup as compared with €94.9 million for protons only (342). Thus, for an additional capital investment of €1 million for HT, proton HTRT could offer the putative thermoradiobiological advantages of ^12^C ion therapy. This cost-effective option deserves serious consideration depending on the outcomes of current trials with proton HTRT (266, 305).

Low recurring costs, the possibility of dose de-escalation, and the lack of additional normal tissue toxicity with HT favor this as a cost-effective treatment. This would be of considerable value to the health administrators and insurance companies if HT is integrated in the clinical practice of oncology.

A better local control along with low treatment related normal tissue morbidity as has been observed in various studies with HT could even pave the way for organ preservation strategies and thus improve the quality of life of patients. Moreover, a therapy that has shown to be effective even in patients who had failed in all other standard treatment options (e.g., recurrent breast cancers) certainly deserves to be evaluated upfront as a part of definitive therapy in *de novo* patients rather than only as a palliative tool in recurrent cases failed to standard forms of treatment.

## Hyperthermia: Tows Analysis

**Figure 8:**
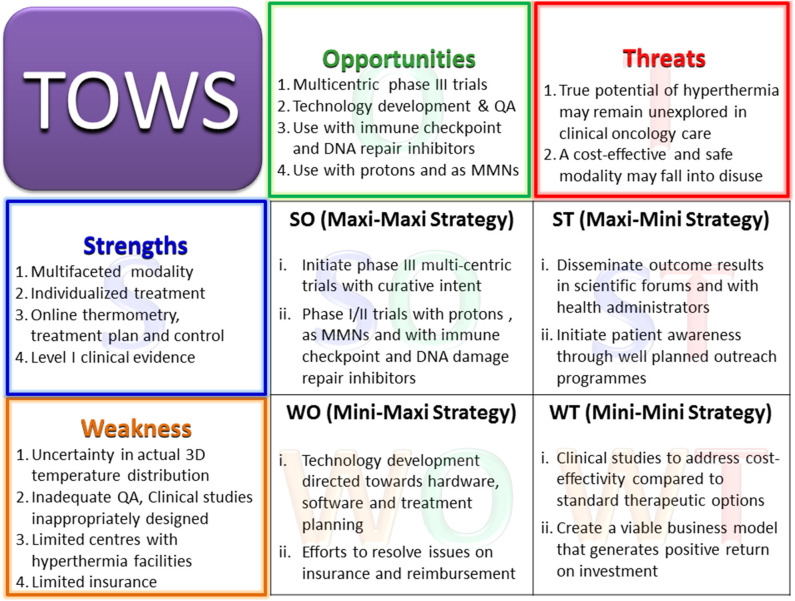
A TOWS analysis to identify key strategies to take advantage of the strength and opportunities while overcoming the weakness and reducing the threats. This could enable integrate hyperthermia in the present clinical armamentarium of oncology care. MMNs, multifunctional magnetic nanoparticles.

A TOWS analysis allows identifying relevant strategies that could facilitate integration of HT in the mainstream oncology therapeutics (Figure 8) (305, 343). These are as follows.

A: “Maxi-maxi” (SO) strategies that use strengths of HT to maximize its opportunities

- Well-designed multicenter phase III clinical trials with adequate patient numbers and quality assurance of heating and temperature monitoring are key requirements. Common treatment protocols endorsed by national or international hyperthermia scientific societies could help new centers to standardize their treatment protocols in accordance with guidelines and assistance from experienced centers. Curative indications where standard therapy yields unsatisfactory outcomes should be preferred to assess the impact of HT on long-term endpoints including both survival and toxicity. These strategies could also include a combined approach with HTRT and/or CT (locally recurrent breast cancers, reirradiation, cutaneous malignancies, bone metastasis, soft tissue sarcoma, LAHNC, LACC, muscle invasive bladder cancer especially for elderly who are not medically fit for surgery/CT, malignant melanoma, locally advanced pancreatic cancers, ano-rectal cancers, and others), with other novel agents like ICI (malignant melanoma, bladder, head, and neck cancers) (110) and PARP1 inhibitors (107, 108, 133, 137–139) in well-designed clinical trials. The list of tumor sites is not exhaustive but is suggestive based on the reported outcomes from various phase II/III clinical trials, pair-wise meta-analysis, and NMA as has been detailed earlier.
- Proton HTRT opens up an exciting cost-effective alternative to ^12^C ion therapy and MMNs, with the potential of magic (nano)bullets, which deserve further investigation in clinical settings. Additionally, phase I/II clinical trials should be initiated with HTRT along with ICI and DNA damage repair inhibitors.

B: “Maxi-mini” (ST) strategies that use strengths of HT to minimize its threats

- The “Threat” that HT, as a cost-effective and a safe modality, may fall into disuse could be overcome by its unique “Strength” as multifaceted modality—a potent radiosensitizer, a chemosensitizer, and an immunomodulator. To the best of our knowledge, presently, none of the therapeutic modalities in oncology possess such a wide range of efficacy and that too without significantly increasing the morbidity and mortality. Thus, a “Maxi-mini” strategy could be used to circumvent the “Threat” by taking advantage of its “Strength.” Consequently, awareness of the favorable clinical outcomes obtained with HT needs to be increased through scientific oncology forums. This would help to reduce unfounded skepticism about the therapeutic benefits of this modality among oncologists unfamiliar with HT and promote its gradual acceptance in clinical care.
- Most oncologists encourage a comprehensive discussion with their patients regarding the proposed treatment to gain informed consent. A well-structured outreach program for the public could apprise them of the therapeutic role of HT. This would enable patients to exercise their choice and explore the feasibility for treatment strategies that include HT.

C: “Mini-maxi” (WO) strategies to minimize weakness of HT by taking advantage of opportunities

- Recent advances and ongoing technological developments in system design, quality assurance, and treatment planning have significantly improved treatment delivery in terms of thermal dose and optimization of synergy with concurrent CTRT. To ensure that HT equipment meets the clinical needs, manufacturers should invest in research and development in consultation with clinicians.
- National HT societies should increase efforts to resolve issues regarding insurance and reimbursement with the governmental agencies, as was already successfully done in a number of countries (281, 282).

D: “Mini-mini” (WT) strategies that minimize weakness of HT and avoids threats

- Cost–benefit and cost-effective analyses are essential given today's spiraling health-care costs. Economic evaluations for HT could provide policy makers with additional information to evaluate the economic viability of HT in comparison with that of other therapeutic interventions. Selection of more costly HT equipment (e.g., with integrated MR guidance) will be determined by control requirements for specific tumor site. The cost-effective assessments should be an integral part of all future randomized clinical trials.
- A viable business model is desirable for all capital cost investment projects, including establishing HT infrastructure. With low recurring costs, a favorable return on investment with HT appears to be realistic.

Thus, the key strategies emerging out of the TOWS analysis that could be adopted to expedite the integration of hyperthermia in clinical oncology practice are as follows:

- Well-designed multicentric phase III trials in tumor sites, especially in those clinical situations where standard therapeutic interventions yield unsatisfactory outcomes (e.g., locally advanced pancreatic cancers) and also in situations where organ preservation approach with HT could add immense value to the quality of life (e.g., soft tissue sarcomas of the extremities and anal cancers).
- Improved quality assurance measures in HT treatments: this includes use of reliable and reproducible operator-independent treatment control procedures based on online planning and online temperature feedback to achieve the desired tumor temperatures.
- Increasing awareness among the oncologists and patients about the potential therapeutic advantages with HT.
- Increasing health insurance and reimbursements highlighting the cost–benefit and cost-effectiveness of integrating HT with existing treatment modalities.

## Conclusion

Hyperthermia is a unique therapeutic modality with multifaceted actions including potent radiosensitization, favorable interaction with a wide range of chemotherapeutic agents, and strengthening of the immunomodulatory response. Evidence from preclinical and clinical studies, supplemented by online temperature monitoring and HT treatment planning, allows a safe and effective personalized treatment. This is borne out of the absence of significantly enhanced acute or late toxicity with HT.

Historically, HT had a mixed profile. Efficacy reported from *in vitro* and *in vivo* studies prompted clinics to adopt HT with RT with reasonable success. However, HT fell into disrepute owing to inadequate heating techniques and temperature monitoring in early trials, combined with poor patient selection. Serious efforts since then addressed the technical lacunae, resulting in effective heating equipment and QA protocols, yielding good clinical results. Further improvement and even better results can be achieved by optimizing the interaction of HT with RT, for example, with optimal timing, various inhibitors of DNA damage repair, ICIs, protons and MMNs, offers exciting new prospects for clinical application. This calls for well-designed phase III randomized clinical trials, as these form the Achilles' heel for widespread acceptance and integration of HT in the routine clinical oncology management.

**Figure 9:**
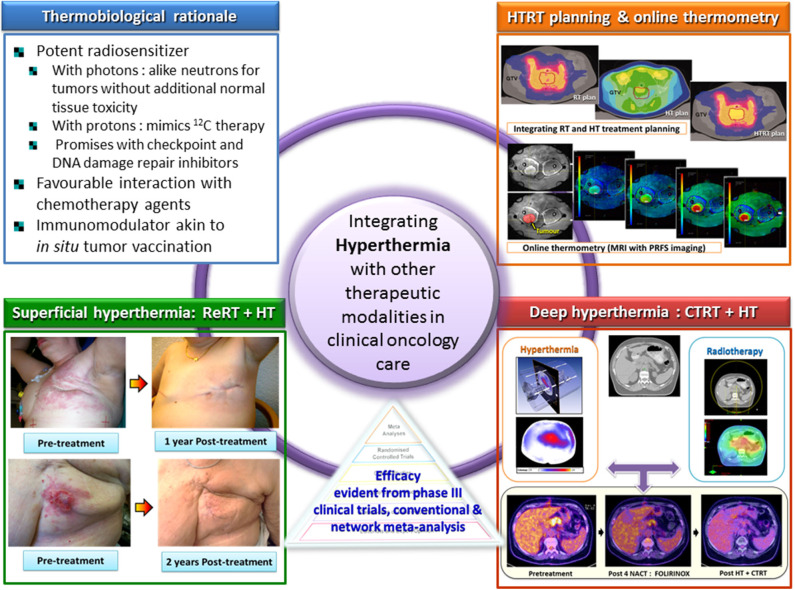
Integration of hyperthermia in clinical practice along with other treatment modalities is supported by its thermobiological rationale and clinical evidences reported from various phase III randomized trials and meta-analysis of various tumors sites. ReRT, reirradiation; HT, hyperthermia; RT, radiotherapy; HTRT, thermoradiotherapy; CTRT, chemoradiotherapy; NACT, neoadjuvant chemotherapy; PRFS, proton resonance frequency shift. The images of RT, HT, and HTRT plans have been modified and reproduced with permission from van Leeuwen et al. (262).

These SWOT and TOWS analyses, reported for the first time for HT, present a critical and systematic appraisal of the present status of HT. The data show that HT facilitates all therapeutic modalities without an increase in acute or late morbidities. Thus, integrating HT in the therapeutic armamentarium would result in a “win–win” situation (Figure 9). All stakeholders need to undertake a coordinated action to promote HT in clinical oncology “with an open mind and a cool head” (13). Otherwise, we risk depriving our cancer patients of a unique modality with a huge potential to improve outcomes without significantly adding acute or late tissue morbidity.

## Author Contributions

ND conceived and designed the study. ND, HK, HC, and UG prepared the manuscript. ND, HK, HC, UG, and SB edited and reviewed the manuscript.

## Conflict of Interest

The authors declare that the research was conducted in the absence of any commercial or financial relationships that could be construed as a potential conflict of interest.
